# Integrating Health and Disability Data Into Academic Information Systems: Workflow Optimization Study

**DOI:** 10.2196/54859

**Published:** 2024-09-04

**Authors:** Abdulrahman Jabour

**Affiliations:** 1 Department of Health Informatics, Faculty of Public Health and Tropical Medicine, Jazan University, Jazan, Saudi Arabia

**Keywords:** disability, health data, student health, health measures, disability data, university setting, university system, student system, academic system, health informatics, health-related, health information, support, well-being, user-centered, data collection, analysis, development, privacy, confidentiality, timely communication, task automation, resources, quality of life, wellness, advisor, advisors, support system, interview, interviews, administrative staff, admin staff, physician, physicians, faculty, student, students, thematic analysis, focus group

## Abstract

**Background:**

Integrating health information into university information systems holds significant potential for enhancing student support and well-being. Despite the growing body of research highlighting issues faced by university students, including stress, depression, and disability, little has been done in the informatics field to incorporate health technologies at the institutional level.

**Objective:**

This study aims to investigate the current state of health information integration within university systems and provide design recommendations to address existing gaps and opportunities.

**Methods:**

We used a user-centered approach to conduct interviews and focus group sessions with stakeholders to gather comprehensive insights and requirements for the system. The methodology involved data collection, analysis, and the development of a suggested workflow.

**Results:**

The findings of this study revealed the shortcomings in the current process of handling health and disability data within university information systems. In our results, we discuss some requirements identified for integrating health-related information into student information systems, such as privacy and confidentiality, timely communication, task automation, and disability resources. We propose a workflow that separates the process into 2 distinct components: a health and disability system and measures of quality of life and wellness. The proposed workflow highlights the vital role of academic advisors in facilitating support and enhancing coordination among stakeholders.

**Conclusions:**

To streamline the workflow, it is vital to have effective coordination among stakeholders and redesign the university information system. However, implementing the new system will require significant capital and resources. We strongly emphasize the importance of increased standardization and regulation to support the information system requirements for health and disability. Through the adoption of standardized practices and regulations, we can ensure the smooth and effective implementation of the required support system.

## Introduction

In recent years, there has been a growing body of research highlighting the profound impact of health and well-being on students’ academic achievements [1]. Numerous studies have documented the significance of maintaining good physical and mental health in order to support students’ overall well-being and optimize their educational outcomes [1-3]

One particularly alarming trend is the increasing prevalence of stress, depression, and anxiety among students. These mental health challenges have been shown to have detrimental effects on students’ physical health and their academic performance [1]. Consequently, it has become essential to address these issues and implement effective strategies to support students’ well-being throughout their educational journey [4].

Furthermore, students with disabilities and special needs form an integral part of university life. Inclusive education is not only a moral imperative but is also mandated by standards and regulatory requirements. However, students with disabilities often face unique challenges that can hinder their full participation in academic and social activities on campus. Navigating the complex landscape of campus resources, overcoming the stigma associated with invisible and physical disabilities, and combating feelings of isolation are just a few of the obstacles that these students encounter [5-7].

In light of these challenges, it is crucial to explore and address the role of health information technologies in supporting the health and well-being of university students. Despite recent advancements in health information technologies, there is a noticeable gap in research focusing on health information and university information systems. These systems play a central role in managing various aspects of university life, including academic support, administrative processes, and campus resources. However, the incorporation of health information into these systems remains relatively unexplored.

Recent studies highlight the vital role of information and communication technologies (ICTs) in supporting students with disabilities in higher education. Fichten et al [8] discuss how, despite generally adequate support on campuses, significant gaps exist in home and e-learning environments. Sharby and Roush [9] propose a decision-making model tailored to the needs of allied health students, emphasizing tailored accommodations and maintaining academic integrity. Stein et al [10] show how a knowledge-based system can effectively support disability accommodations under the Americans with Disabilities Act (ADA), enhancing institutional support and compliance. These insights reveal an urgent need for universities to improve ICT integration and support to foster inclusivity and aid students with disabilities. Furthermore, these gaps underline the necessity for better adaptive technology access off campus and increased faculty training in disability accommodation practices.

Understanding the potential benefits and challenges associated with integrating health information into university information systems is essential for enhancing the overall support and well-being of students. By leveraging the capabilities of modern information systems, universities can better address the unique health needs of their student population, promote inclusivity, and empower students to achieve their full academic potential. The analytical decision-making model proposed by Sharby and Roush [9] offers a comprehensive theoretical framework for integrating health information into university information systems. This model’s systematic approach to accommodating diverse needs can be adapted to evaluate how health data are integrated into university information systems, ensuring these systems meet the varied requirements of students, faculty, and staff effectively. By applying this model, universities can better identify gaps and implement tailored solutions that enhance accessibility and functionality within their health information ecosystems.

Therefore, the aim of this study is to investigate the current state of health information integration within university information systems and provide design recommendations. By examining the existing system and stakeholders needs, this study seeks to shed light on gaps and opportunities for integrating health information technologies into university systems. The findings of this research will contribute to the growing body of knowledge in health information technologies and inform the development of strategies and interventions aimed at improving student well-being in higher education settings.

## Methods

**Figure 1.**
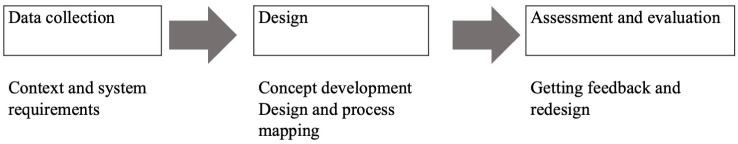
Summary of the study design steps describing each phase.

In this study, we used a user-centered approach to integrate health data into the university information system [11,12]. The methodology consisted of data collection, design, and assessment and evaluation [13] (Figure 1). The following subsections describe each phase in detail.

### Data Collection

To gather comprehensive insights and users’ requirements for the system, a series of interviews and focus group sessions were conducted with stakeholders. The interview phase involved engaging with a diverse group of participants, including 3 academic advisors, 3 administrative staff working on student affairs related to health and academic performance, 2 physicians/health counselors, 3 health professionals, and 4 teaching faculty members. The participants’ experience ranged from 6 to 13 years. These stakeholders were chosen based on their expertise and previous involvement in activities and cases related to students’ health information. In the methodological design of this study, we considered content saturation to be achieved within the 15-participant sample, as no additional unique insights were observed beyond this point. Consistent with findings from the usability testing literature, studies such as that by Faulkner [14] have shown that increasing the sample size from 5 to 10 participants can capture up to 95% of usability issues, thus providing a robust basis for our sample size decision and ensuring comprehensive data collection for system evaluation.

Additionally, 2 focus groups were conducted with 10 students. All students were undergraduates, and their age ranged from 19 to 24 years. The aim of these focus groups was to understand students’ perspectives and needs.

Before the interviews, participants were given introductory information about the research project. Participation was voluntary, and they were allowed to skip questions, withdraw, or end the interview at any time without any conditions. No compensation was offered for participation. Prior to initiating the interview, participants were briefed on the study’s scope and provided their informed consent. The interviews were unstructured, and probing questions were introduced as needed (Multimedia Appendix 1). The main areas covered during the interviews are described in the following sections.

#### Background and Context

We sought to understand the stakeholders’ roles and responsibilities, as well as their relationship to the student health information system being developed.

#### Goals and Objectives

Our aim was to identify the stakeholders’ primary goals, objectives, and desired outcomes related to the system. This included understanding what they hoped to achieve or improve through the use of the system.

#### System Requirements

We gathered requirements from stakeholders regarding the desired functionalities, features, and capabilities of the health information system. This included understanding the specific data elements, information flows, and interactions that are important to stakeholders.

#### Use Cases and Workflows

We examined the typical use cases and workflows of stakeholders within the context of the health information system. This involved understanding how stakeholders interact with the system, the sequence of steps they take, and any dependencies or constraints they encounter.

#### Data Privacy and Confidentiality

This involved discussing stakeholders’ concerns and requirements related to data privacy, confidentiality, and compliance with relevant regulations. This included understanding their expectations for data protection, access control, and confidentiality.

#### Initial Phase and Workflow Analysis

During the initial phase of data collection, the focus was on gathering system requirements, as well as understanding the use cases, flow of information, restrictions, and other pertinent factors. The interviews and focus groups were guided by open-ended questions and prompts to encourage participants to provide detailed insights and suggestions.

The data collection process also included an analysis of the existing workflow and an artifact analysis. The artifacts included the existing forms, screenshots, templates, instructions, and guidelines used for data gathering and communication.

### Design

Following the data collection phase, thematic analysis was conducted to identify patterns, common themes, and user requirements [15]. This analysis informed the design phase, where a conceptual design of the workflow was developed. The design process involved translating the gathered requirements into system functionalities and a workflow. In addition, an artifact analysis was conducted to examine existing forms, templates, and guidelines related to the workflow. This analysis involved systematically reviewing and coding these documents to identify recurring themes, inefficiencies, and opportunities for improvement. The artifacts were evaluated based on criteria such as clarity, efficiency, and alignment with the workflow.

To establish a holistic approach, the design phase was informed by the systems model of Bowman and Marzouk [16] for implementing the ADA in higher education. Their model, based on general systems theory, encompasses a comprehensive structure with input, throughput, and output subsystems. By adopting this systems model, the workflow design addressed core components such as accessibility, learning resources, and quality control.

To enhance the design methodology, insights were incorporated from previous studies to provide a broader understanding of user requirements and system functionalities. Fichten et al [8] contributed valuable information on variables related to how well the ICT needs of students with different disabilities are being met at institutions of higher education, at home, and in e-learning contexts. Their work also explored the disciplines and programs pursued by students with varying disabilities and the specialized ICTs used. Stein et al [10] provided a knowledge-based system design to assist university administrators in meeting the requirements of disability-related legislation. This ensured that the proposed system was comprehensive and aligned with compliance standards. Finally, Sharby and Roush [9] delivered an analytical decision-making model for addressing the specific needs of allied health students with disabilities, offering accommodations that are both practical and inclusive.

### Assessment and Evaluation

Once the conceptual design was drafted, participants were presented with the proposed design and concept. Their feedback and suggestions were collected to ensure their perspectives were considered in the development process. An iterative and agile approach was adopted to incorporate the feedback received from participants.

### Ethical Considerations

The study was approved by the ethical review committee at Jazan University (REC-45/11/1100). Participation for both staff and students was entirely voluntary, and no compensation was provided to participants. Participants were allowed to withdraw at any time without any conditions. Informed consent was obtained and all data collected were anonymized to ensure the privacy and confidentiality of participant information.

## Results

### Current System

Based on our analysis, we have identified several systemic challenges and shortcomings in the existing system, which are summarized in the following sections.

#### Variation and Inaccuracy of Student Information

We found that the information entered by students varies in nature and is often incomplete or inaccurate. This creates additional work for university staff and committees, who must gather more information and assess individual cases. Students tend to understate or overstate their conditions, further complicating the process.

#### Absence of University Health Care Center Representation

Currently, the university health care center is not involved in the process. When students require medical reports for absences or other requests, they upload the report from their accounts. This lack of integration creates inefficiencies and delays in the system.

#### Exclusion of Academic Advising Unit

The academic advising unit is not part of the process, which means that advisors cannot proactively identify or address students’ needs unless a request is specifically referred to them by a staff member. This disconnect hinders effective support and guidance for students.

#### Neglect of Disability-Related Accommodations and Student Needs During Registration

The current system does not adequately consider disability-related accommodations and students’ individual needs during the registration and enrollment process. There is no distinction made between different classrooms or labs, nor is there information on the availability of relevant resources. This oversight can lead to accessibility issues and hinder students’ academic experience.

### Design Considerations

Our results identified several requirements that need to be taken into consideration for integrating health-related information into the student information system. We categorized these needs into the following 4 categories: privacy and confidentiality, timely communication, workload and automation, and disability resources.

#### Privacy and Confidentiality

Our results revealed that students’ health information requires a particular degree of privacy. Disseminating such information indiscriminately among all stakeholders encompassed within the system or involved in the process may not align with best practices. To overcome this challenge, we proposed a system that empowers the student advising unit. This system allows for the evaluation of received information, assessment of specific requirements, and addition of labels, tags, or notes detailing students’ needs. This will enable the effective safeguarding of the privacy of students. Additionally, in scenarios where medical reports are needed by the university administration or external committees for request assessments or legal matters, exceptions can be formulated to ensure appropriate sharing.

#### Timely Communication

Our interviews with stakeholders highlighted recurrent instances where students had a low attendance rate due to health conditions that required regular hospital visits, such as sickle cell anemia or cancer. We found that these students were sometimes reluctant to address their needs openly. Many were only discovered during the consultation session with the student’s advisor after being called for a low attendance rate. Providing detailed health information about students early on in their academic journey can help student advisors support them better.

#### Workload and Automation

In our study, we discovered that a significant portion of the tasks related to managing health information were being carried out manually. This manual approach posed several challenges, including inconsistencies and variations in practice, as well as an increased workload for staff members. To address these issues and streamline the process, the proposed workflow emphasizes the importance of automating certain activities. For example, by implementing automation, we can effectively match students’ needs to appropriate classrooms; ensure the availability of necessary equipment and infrastructure; send timely notifications for attention; calculate students’ health measures, such as stress, depression, and anxiety; and provide tailored advice and recommendations to support students in overcoming difficulties. By leveraging automation, we can alleviate the burden on staff members and enhance the overall efficiency and effectiveness of managing health information for a large number of students.

#### Disability Resources

While some university systems contain sections for disabilities, information regarding specific health conditions or academic needs are not always incorporated into academic information systems [17]. Moreover, the inclusion of disability information is not fully streamlined or integrated in the workflow, such as by automated flagging and registering of students with a disability that need specialized resources and accommodations in unequipped rooms or laboratories. These needs span from relatively minor adjustments such as left-handed tables to conditions that may need wheelchair accessibility or technological aids for visual and auditory impairments. The integration of health conditions within the academic information system is recommended to empower administrators and registrars in the seamless allocation of classrooms and laboratories suitably equipped to address these distinctive needs.

### Data and Information

Based on the interviews, it is evident that the design of student information systems needs to consider various aspects of students’ health. In this study, we categorized the types of information required into 3 categories, which we describe in the following sections.

#### Information Related to Disabilities and Special Needs

This category pertains to students with disabilities; examples include physical and mental disabilities, as well as vision and hearing impairments. It is crucial to consider the specific needs of these students to ensure an inclusive educational environment. Understanding their disabilities and special needs allows educational institutions to provide appropriate accommodations and support services, promoting equal opportunities for learning and participation.

#### Information Related to Health and Chronic Conditions

This category encompasses both preexisting chronic health conditions that may impact a student’s educational journey and health-related events that might affect their attendance. Our results suggest that students with chronic health conditions face unique challenges in maintaining academic progress, managing their health, and navigating the educational system. Incorporating information about these conditions into students’ information systems enables educational institutions to provide interventions, accommodations, and support tailored to individual needs.

#### Information About Continuous Health Measures

While the previous 2 categories focus on students with specific health issues, we propose that continuous health measures should be included for all students. These measures may involve assessing stress, anxiety, and depression levels. This can be very valuable for monitoring and addressing mental health concerns in educational settings to enhance overall well-being, academic performance, and retention rates. Collecting data on continuous health measures can facilitate early intervention and the implementation of appropriate support systems.

### Workflow Description

To design the workflow, we separated the process into two different processes: (1) a health and disability system and (2) quality of life (QoL) and wellness measures (Table 1).

**Table 1.** Description of the data needed and frequency of collection.

Process and data		Collection frequency
**Health and disability system**		
	Information related to disability and special needs	Entered once at the beginning of enrollment and updated as needed
	Information related to health and chronic conditions (preexisting chronic health conditions)	Entered once at the beginning of enrollment and updated as needed
	Information related to health and nonchronic conditions (health-related events)	Entered as needed
**QoL^a^ and wellness system**		
	Information about continuous QoL and wellness measures including stress, depression, and anxiety	Systemic collection, once every semester

a QoL: quality of life.

### Health and Disability System Workflow

**Figure 2.**
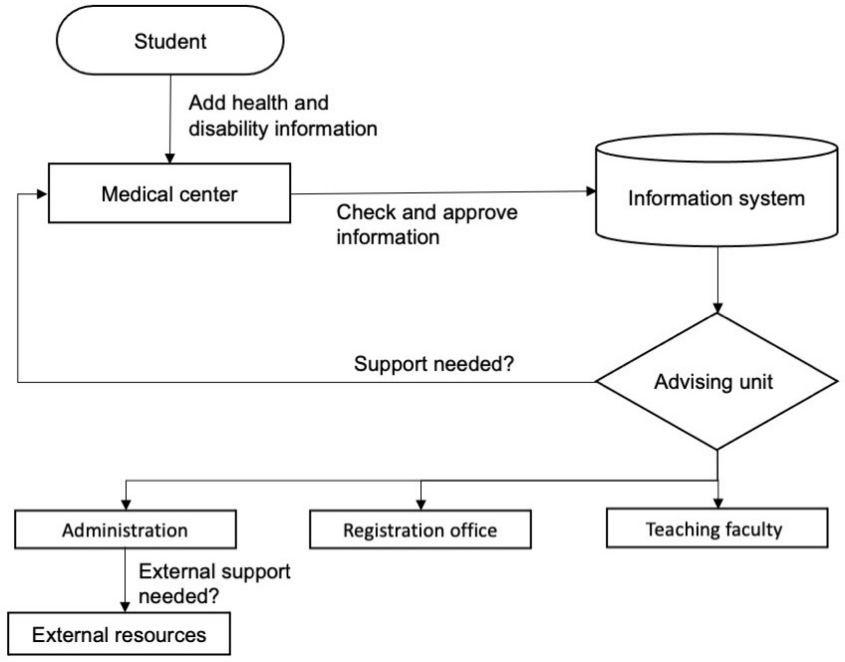
Health and disability system workflow.

Information related to disability and special needs are entered by students for health center review and approval (Figure 2). The university health center approves and enters the students’ health-related information. Based on the type of disability selected, the system suggests a classroom or laboratory based on the availability of necessary accommodations that the student may need, including academic, technological, or administrative needs. The advising unit receives notification of registrations that have a mismatch between disabilities and resources for further attention. If needed, the student advising unit identifies the necessary accommodation that a student may need and sends messages to communicate with the registration office, administration, and teaching faculties.

### QoL and Wellness System Workflow

**Figure 3.**
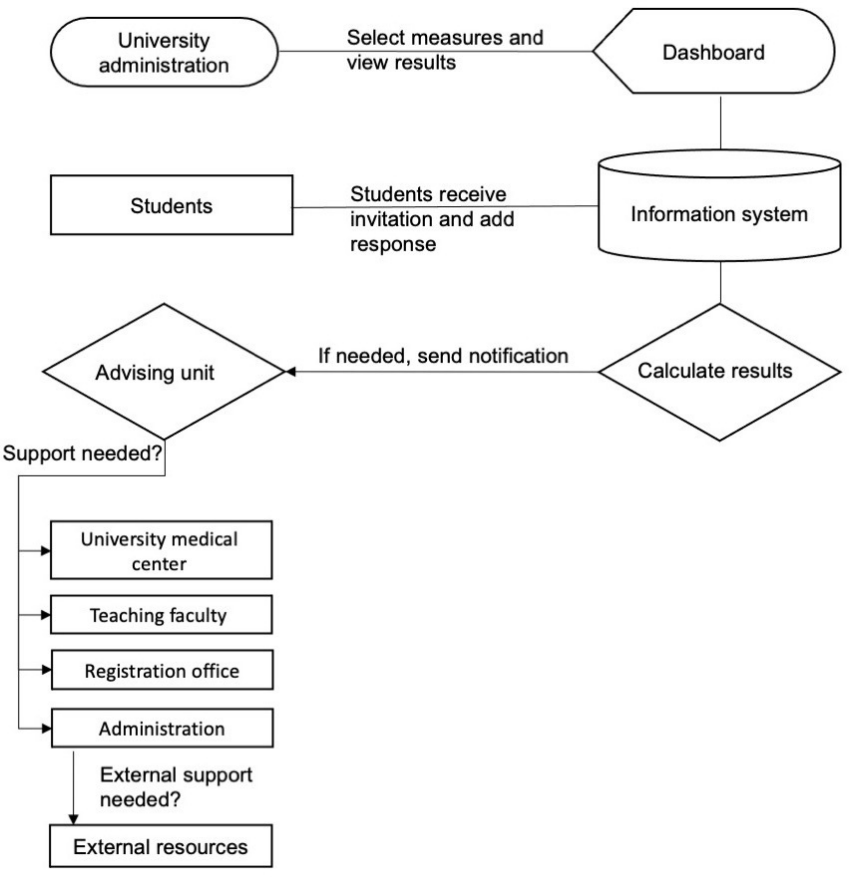
Quality of life and wellness system workflow.

The university administration selects the health measures they are interested in and specifies the frequency of measurement (Figure 3). Students receive the invitation and complete their responses. The university administration uses the dashboard to view the results, including summary information and a data overview. Responses with low scores that needs attention are identified by the system, triggering a notification that is sent to the advising unit for attention. The advising unit reviews the triggered student response and takes the necessary action.

## Discussion

### Principal Findings

In this study, we investigated the current status of integrating health and disability information within university systems, identified the existing gaps, and provided design considerations. Using a user-centered design approach, we assessed the impact of these design considerations on students’ success and inclusion. Our findings revealed several challenges. These challenges include privacy concerns, underidentification of stress, anxiety, and depression, and reluctance among students with hidden disabilities to disclose their conditions due to fear of stigma and discrimination. Our user-centered design approach addressed these issues by emphasizing the role of advising units in maintaining privacy and facilitating support. Additionally, the design focused on resource allocation to meet the diverse needs of students with various disabilities, ensuring accessibility and adaptive learning support. By incorporating health and disability information into university systems, the proposed design aims to enhance inclusivity, improve resource allocation, and promote better academic outcomes for all students. In the subsequent sections we discuss these findings and provide recommendations for future work.

Incorporating health and disability information is crucial for promoting students’ academic success. However, effectively integrating this information into existing systems remains challenging. To address this challenge, there is a need to incorporate the use of health and disability information into the academic process and daily activities. The appropriate inclusion and use of health and disability information within university information systems offers a promising approach to integrate these data into daily tasks and processes, leading to enhanced effectiveness and improved outcomes. In our study, we adopted a user-centered approach to design the integration of health and disability information into university information systems. This approach prioritizes the needs and perspectives of users, ensuring that the system is tailored to their requirements and preferences. By considering the user experience and incorporating user feedback throughout the design process, we designed a system process that effectively incorporates health and disability information, ultimately enhancing its usability and impact on student success.

Our study uncovered many challenges in the existing system. Among these challenges was the privacy and confidentiality of students’ health information. While obtaining knowledge about students’ health events is essential for providing adequate support and accommodation, some students were reluctant to share their health information due to concerns about privacy breaches, stigma, or fear of discrimination.

Another significant challenge was the underidentification and underdisclosure of psychological conditions such as stress, anxiety, and depression among students. Many students perceive stress and anxiety as a normal part of the academic journey and may not seek professional help to differentiate between healthy stress and unhealthy stress. The systematic collection of stress, anxiety, and depression data via validated measures offers great potential for timely interventions and support. Furthermore, students with hidden disabilities often encounter barriers that hinder their university experiences [18]. Due to a desire to avoid the label of “disability,” many students with invisible disabilities choose not to access the supports they are entitled to, which can hinder their academic success [18,19].

To address these challenges, the proposed design emphasizes the role of student advising units in facilitating support while maintaining students’ privacy. Advising units can play a crucial role in creating a safe and supportive environment where students feel comfortable sharing their health information and accessing the necessary support services [20,21]. By establishing trust and providing guidance, advising units can help students navigate the system while respecting their privacy needs.

One of the key use cases found during interviews was the allocation of resources to meet the diverse requirements of students with disabilities. It was evident that different types of disabilities present unique needs and challenges. For instance, students with hearing or vision impairments may require special technological resources to facilitate their learning experience [22,23]. On the other hand, students with physical disabilities require accessibility measures, such as wheelchair access, that should be available in various locations across the campus.

Furthermore, the interviewees emphasized the importance of addressing the adaptive learning needs of students. This includes considering students with conditions such as cognitive disabilities and other health conditions that may impact their attendance [21]. While the specific needs may vary in each case, being aware of these needs is crucial for effective preparation and support.

By incorporating health and disability information into the university information system, administrators and faculty members can gain a comprehensive understanding of the diverse requirements of students. This knowledge enables them to allocate resources more effectively and provide appropriate support, ultimately promoting inclusive education and enhancing student success [20,21].

In order to ensure comprehensive inclusion and accessibility, it is crucial for accreditation bodies and policy frameworks to address the needs of individuals with disabilities. While physical accommodations such as wheelchair access, left-handed writing tables, and disability-friendly bathrooms are typically addressed in these standards, there appears to be a significant gap in incorporating disability-related information into IT standards and guidelines. Despite the growing reliance on IT in various domains, there is a lack of explicit guidance on how to handle disability-related information and promote its integration into IT systems. This oversight is concerning given the potential of IT to enhance accessibility and empower individuals with disabilities. By failing to address the inclusion of disability-related information, accreditation bodies and policy frameworks miss an opportunity to ensure equal access and participation for all.

While the current academic accreditation includes requirements related to students’ disabilities in the physical environment, such as wheelchair access, it does not address requirements related to student information systems [24]. To address this gap, it is essential for accreditation bodies and policy guidelines to recognize the importance of incorporating disability-related information into IT standards. This could involve establishing guidelines for designing accessible user interfaces, ensuring compatibility with assistive technologies, and promoting the provision of alternative formats for individuals with sensory impairments. Additionally, the safe handling and protection of disability-related data should be emphasized to safeguard the privacy and confidentiality of individuals with disabilities.

By actively addressing the inclusion of disability-related information within IT standards, accreditation bodies and policy frameworks can contribute to a more inclusive and accessible society. This proactive approach will enhance the usability and effectiveness of IT systems, promoting equal opportunities and empowering individuals with disabilities to fully participate in various aspects of life.

### Limitations and Future Work

The proposed design offers potential solutions to address the issue of late discovery of health-related cases through systematic data collection and communication with student health clinics. However, challenges persist due to students’ reluctance to disclose their health information out of concerns related to privacy breaches, stigma, and fear of discrimination.

The potential side effects of health information disclosure are a crucial area that requires careful attention. The threat of privacy breaches, stigma, and discrimination needs to be carefully assessed, and risk management strategies should be established to ensure the protection of students’ privacy and prevent any negative consequences.

Although the design process described here incorporated user input, our study was limited to a relatively small sample size. For future work, we recommend prototype usability testing and evaluation of the redesigned model with a larger and more diverse group of users. This would provide a broader range of perspectives, facilitating further evaluation and improvements to the design. In addition, interpretation of our findings should be approached with caution, as the homogeneity of the participant pool, all drawn from a single institution, may limit the generalizability of our results

Future studies should also explore strategies to address privacy concerns, enhance security measures, and develop clear communication strategies to alleviate students’ fears regarding the disclosure of their health information. Additionally, assessing the impact of the proposed design on student outcomes, such as academic performance and well-being, would provide valuable insights into its effectiveness and usefulness.

### Conclusion

In conclusion, our study has identified several shortcomings in the current process of handling health and disability data. Based on our findings, we recommend implementing a new, redesigned process that addresses these issues. Effective coordination among stakeholders is crucial for the success of this process, and redesigning the university information system is essential for organizing and streamlining the workflow.

While implementing these changes will require significant resources and commitment, we strongly advocate for increased standardization and regulation to support information system requirements related to health and disability. By adopting standardized practices and regulations, we can ensure that the necessary information system is in place to effectively support these needs.

## Supplementary material

10.2196/54859 Multimedia Appendix 1 Interview script.

## Abbreviations

ADA: Americans with Disabilities Act
ICT: information and communication technology
QoL: quality of life
