# Supplementary material for: Integrating Health and Disability Data Into Academic Information Systems: Workflow Optimization Study
Source: JMIR Hum Factors. 2024 Sep 4;11:e54859. doi: 10.2196/54859 (PMC11440573; doi:10.2196/54859)
Supplement: Multimedia Appendix 1 [file humanfactors-v11-e54859-s001.docx]

**Appendix 1**

**Interview script**

**1. Background Context of Use**

- **Main Question:**
  Can you describe your role and responsibilities in relation to the student health information system?
- Walk me through the typical process, step by step.
- What information do you need to add for this step of the process? (Repeat for each process if needed)
- Have you encountered a time in which it did no work as intended?
- (If yes) What other workaround do you do if it did not go as it supposed to do?

**Probing Questions:**

- How do you interact with the current system in your daily activities?
- How do you interact with the current system in to preform (certain task)?
- In what ways has the system impacted your tasks?

**2. Goals and Objectives**

**Main Question:**
What are your primary goals and objectives for using the student health information system?

**Probing Questions:**

- What improvements or outcomes do you wish to see in this system?
- Are there specific problems or challenges you encounter or want to address with this system?

**3. System Requirements**

**Main Question:**
What functionalities and features do you consider essential for the student health information system?

**Probing Questions:**

- Could you specify the types of data and information flows that are critical for your operations?
- Are there any existing capabilities you find lacking or would like to see enhanced?

**4. Use Cases and Workflows**

**Main Question:**
Can you walk me through an example or scenario?

**Probing Questions:**

- What are the steps you typically follow when using the system?
- Are there any points in the workflow where you encounter delays or challenges?

**5. Data Privacy and Confidentiality**

**Main Question:**
Do you have any concerns regarding data privacy and confidentiality within the health information system?

**Probing Questions:**

- How do you expect the system to manage and protect sensitive information?
- Are there specific regulations or compliance requirements that the system needs to meet?

**General Probing Techniques**

- **Clarification:** "Could you explain further what you mean by..."
- **Examples:** "Could you provide an example of what you just mentioned?"
- **Comparison:** "How does this compare with other systems you have used if any?"
